# IPUMS Multigenerational Longitudinal Panel

**DOI:** 10.23889/ijpds.v9i5.2871

**Published:** 2024-09-18

**Authors:** Dana Riley, Eric De Prophetis, Mayuri Mahendran, Geoffrey Hynes, Ezra Hart, Rukayyah Akinnibosun, Keith Denny

**Affiliations:** 1 Canadian Institute for Health Information

The objective of this analysis was to examine variations in rates of hospital harm (HH), an important indicator of patient safety events, across population subgroups in Canada.

We conducted a descriptive study of HH in provinces and territories using three years (2016 to 2018) of CIHI’s Discharge Abstract Database (DAD) linked to the 2016 Canadian Census, also known as Statistics Canada’s Canadian Census Health and Environment Cohorts (CanCHEC). We investigated the influence of sociodemographic variables not generally available in hospital administrative data.

We calculated age-standardized rates (ASR) per 100 hospital discharges and 95% confidence intervals for HH, stratified by sex, income (area and individual), racialized group, education, immigration status, language, disability status, social and material deprivation, and geography (urban vs. rural/remote).

Despite males experiencing higher rates of harm (ASR 4.36 vs. 4.23), overall females experienced 54% of all HH events, with an average age of 64 (vs age 49 for those discharged without a HH event). For neighbourhood income quintile, the highest rates of HH were observed in the lowest quintile (4.08, 4.04 – 4.11). The rate of HH decreased as neighbourhood income increased. Patients from rural/remote neighbourhoods had lower rates of HH (3.7, 3.66-3.73) than those living in urban neighbourhoods (4.47, 4.44-4.49). Additional equity-stratified results will be presented.

Our analysis revealed that marginalized populations were more likely to experience a harmful event during their hospital stay. Measuring and monitoring inequalities in patient safety provides health systems with information to better understand, target, and evaluate healthcare quality improvement initiatives.

